# Effectiveness and cost-effectiveness of cognitive behavior therapy-enhanced compared with treatment-as-usual for anorexia nervosa in an inpatient and outpatient routine setting: a consecutive cohort study

**DOI:** 10.1186/s40337-021-00526-1

**Published:** 2022-01-06

**Authors:** Elske van den Berg, Daniela Schlochtermeier, Jitske Koenders, Liselotte de Mooij, Margo de Jonge, Anna E. Goudriaan, Matthijs Blankers, Jaap Peen, Jack Dekker

**Affiliations:** 1 Novarum Center for Eating Disorders and Obesity, Laan van de Helende Meesters 2, 1186 AM Amstelveen, The Netherlands; 2 Research Department, Arkin Mental Health Institute, Klaprozenweg 111, 1033 NN Amsterdam, The Netherlands; 3 Department of Psychiatry, Amsterdam UMC, University of Amsterdam, Amsterdam, The Netherlands; 4 Faculty of Behavioral and Movement Sciences, Vrije Universiteit, Amsterdam, The Netherlands

**Keywords:** Anorexia nervosa, Cognitive behavior therapy-enhanced, Treatment-as-usual, Effectiveness, Cost-effectiveness

## Abstract

**Background:**

For anorexia nervosa, firm evidence of the superiority of specialized psychological treatments is limited and economic evaluations of such treatments in real world settings are scarce. This consecutive cohort study examined differential (cost-)effectiveness for adult inpatients and outpatients with anorexia nervosa, after implementing cognitive behavioral therapy-enhanced (CBT-E) throughout a routine setting.

**Methods:**

Differences in remission, weight regain and direct eating disorder treatment costs were examined between one cohort (*N* = 75) receiving treatment-as-usual (TAU) between 2012–2014, and the other (*N* = 88) CBT-E between 2015–2017. The economic evaluation was performed from a health care perspective with a one-year time horizon, using EDE global score < 2.77, the absence of eating disorder behaviors combined with a BMI ≥ 18.5, as effect measure. Incremental cost-effectiveness ratios were calculated and cost-effectiveness planes and cost-effectiveness acceptability curves were displayed to assess the probability that CBT-E is cost effective compared to TAU.

**Results:**

Using direct eating disorder treatment costs in the cost-effectiveness analysis, the cost-effectiveness plane of the base case scenario for all patients indicated a 84% likelihood of CBT-E generating better health gain at additional costs. The median ICER is €51,081, indicating a probable preference for CBT-E (> 50% probability of cost-effectiveness) assuming a WTP of €51,081 or more for each additional remission, On remission, no difference was found with 9.3% remission during TAU and 14.6% during CBT-E (*p* = .304). Weight regain was higher during CBT-E (*EMD* = 1.33 kg/m^2^, *SE* = .29, 95% CI [0.76–1.9], *p* < .001).

**Conclusions:**

In this mixed inpatient and outpatient cohort study, findings indicate a probability of CBT-Ebeing more effective at higher costs. These findings may contribute to the knowledge of effectiveness and cost-effectiveness of specialized psychological treatments.

**Plain English Summary:**

In this study, the effectiveness and treatment costs of a specialized psychological treatment for adult clients with anorexia nervosa were compared with a regular, non-specialist treatment. One group of inpatients and outpatients did receive non-specialist treatment, the next group of inpatients and outpatients received CBT-E, a specialized treatment, later on. CBT-E is recommended for clients with bulimia and with binge eating disorder, for clients with anorexia nervosa it is less clear which specialized psychological treatment should be recommended. Results indicate that at end-of-treatment, CBT-E was not superior on remission. When looking at weight regain, CBT-E seemed superior than the treatment offered earlier. Economic evaluation suggests that CBT-E generates better health gain, but at additional costs. This study contributes to the knowledge on the effectiveness and treatment costs of psychological treatments, as they are offered in routine practice, to adults with anorexia nervosa.

**Supplementary Information:**

The online version contains supplementary material available at 10.1186/s40337-021-00526-1.

## Background

Anorexia nervosa is a psychiatric disorder associated with a poor prognosis. For adults with anorexia nervosa, evidence is lacking for prioritizing one specialized treatment over the other, or even for prioritizing specialized over non-specialized treatments [1, 2]. Furthermore, with regard to regaining weight or with regard to reducing anorexia nervosa psychopathology, no conclusive recommendations can be made on an optimum treatment setting [3, 4], even though guidelines agree on hospital admission in case of high medical or psychiatric risk [4]. Individual eating-disorder-focused cognitive behavioural therapy is one of the specialized psychological treatments for adults recommended by NICE [5]. For underweight patients, Fairburn et al. [6] developed an enhanced eating-disorder-focused individual cognitive behavior therapy (CBT-E *Underweight*), an adaption of CBT-E for normal weight eating disorder patients. Effectiveness studies showed that CBT-E *Underweight* can be delivered in both routine outpatient practice [7–12] and inpatient practice [13]. Despite these findings, implementation of recommended specialized treatments in routine practice lag, especially in inpatient settings [14].

Published CBT-E *Underweight* effectiveness studies involved a limited number of therapists and, in most outpatient studies, patients with severe underweight were excluded [7, 10, 11]. Besides, as the involved outpatient services were unable to offer inpatient care, in case of hospitalization, CBT-E interventions were discontinued.

Examining cost-effectiveness of recommended treatments next to effectiveness, is valuable and may contribute to decision making on what treatment best to offer in clinical practice, but for anorexia nervosa, they are rarely done [15, 16]. An economic evaluation on adolescents with anorexia nervosa suggests that specialist outpatient services have a higher probability of being cost-effective than both general services and inpatient services [17]. Until now, there has been only one economic evaluation of CBT-E *Underweight*; Egger et al., examined the cost-effectiveness of focal psychodynamic psychotherapy, treatment-as-usual (TAU) and CBT-E for outpatients. They found that, employing weight gain as effect measure, CBT-E *Underweight* was dominant compared with TAU, although differences in weight regain between the conditions were negligible [18].

The aims of the present consecutive cohort study were to examine (1) effectiveness and (2) cost-effectiveness of CBT-E *Underweight,* offered to all referred adult anorexia nervosa inpatients and outpatients who are significantly underweight (*N* = 88), receiving treatment between 2015 and 2017, compared with treatment-as-usual offered to all referred inpatients and outpatients (*N* = 75) between 2012–2014. We hypothesized that, compared to treatment-as-usual, implementing CBT-E *Underweight* would lead to an improvement in effectiveness and in cost-effectiveness.

To our knowledge, this is the first CBT-E (cost)-effectiveness study including all referred, mixed inpatients and outpatients, with a BMI between 12.5 and 17.5, during a two-and-a-half year period.

## Method

In this nonequivalent control group pretest–posttest study, differential (cost-)effectiveness was examined between two cohorts of adult, eating disorder outpatients and inpatients, with a body mass index (BMI: kg/m^2^) between 12.5 and below 17.5. The first cohort received treatment-as-usual (TAU), and started and end ended treatment between July 1, 2012 and December 31, 2014 (*N* = 75). The second received CBT-E and started and ended treatment between July 1, 2015 and December 31, 2017 (*N* = 88), after implementing CBT-E throughout all treatment settings. Hence, a six months transition period between both cohorts was constructed in order to prevent possible contamination of TAU with newly learned CBT-E interventions.

### Setting and participants

Both cohorts consisted of consecutive referrals by general practitioners or general hospitals to a routine eating disorder center, offering outpatient and, if indicated, inpatient treatment to patients over 18 on a voluntary basis. All included patients met DSM-IV [19] or DSM-5 [20] criteria for anorexia nervosa or other specified eating disorder similar to anorexia, as assessed by a clinical psychologist or psychiatrist. Exclusion criteria were immediate medical risk requiring medical hospitalization, BMI below 12.5, the presence of an interfering psychotic disorder or cognitive impairment. In addition, since the intake procedure involves up to three contacts, patients with under four contacts were excluded.

The authors assert that all procedures contributing to this work comply with the ethical standards of relevant national and institutional committees on human experimentation and with the Helsinki Declaration of 1975, as revised in 2008. All procedures involving patients were approved by The Dutch Central Committee on Human Research; informed consent, as part of routine service evaluation, was obtained from all patients.

### Assessment

Assessment points were at start and end of treatment. Eating disorder attitudes and behaviors were measured with the Dutch self-report Eating Disorder Examination-Questionnaire (EDE-Q 6.0; [21]), which has good psychometric properties [22]. General psychopathology was measured with the Dutch self-report Depression Anxiety Stress Scale, which has good psychometric properties (DASS-21; [23, 24]).

As primary outcome variable remission was used. Remission is defined as reaching BMI ≥ 18.5, the World Health Organization cut-off point for healthy weight, combined with achieving an EDE-Q global score under one *SD* above community mean, i.e. under 2.77 (UK norms, used for comparison; [25], and the absence of binge/purge behaviours as reported on EDE-Q.

Re-nourishment is not a psychological therapy in itself, but as starvation is a maintaining factor in anorexia nervosa, nutritional changes appear to be necessary for psychological treatments to be effective [26]. Therefore, weight regain was used s secondary outcome measure.

For the cost-effectiveness analyses, the proportion of patients achieving remission was used as effect measure.

### Interventions

#### Treatment-as-usual during July 1th 2012–December 31th 2014

Treatment during the TAU period consisted of regular medical care, restoring underweight through providing supervised meals and behavioral interventions, psychoeducation, psychomotor therapy interventions focusing on body awareness and movement behavior and an eclectic psychological approach of cognitive, behavioral, schema focused and psychodynamic interventions. TAU was delivered in outpatient, day-care and/or inpatient units. Outpatient treatment was preferably group based; day-care was offered to patients with a BMI between around 16.5 and 17.5; inpatient treatment was offered to patients with a BMI below 16.5, and usually lasted until healthy weight was reached. During TAU, structured supervision was not offered.

#### CBT-E during July 1th 2015–December 31th 2017

Outpatients were offered CBT-E for Underweight Patients, a variant of the 20-week version of CBT-E (CBT-E *Underweight),* an individual, outpatient treatment, originally designed by the developers for adult patients who are underweight (those with anorexia nervosa or underweight forms of eating disorders NOS) with a minimum BMI of 15, but in this study offered to all outpatients regardless their degree of underweight. Treatment takes up to about 40 sessions; in accordance with CBT-E guidelines [6], the number of sessions depends on the degree of underweight. CBT-E *Underweight* consists of three phases, the first phase aims at preparing for change, the second at regaining weight and tackling eating disorder pathology, the last phase at developing personalized relapse prevention skills.

CBT-E favors outpatient treatment and states that restoring underweight can take place, when patients are psychiatrically and/or somatically stable, within an outpatient setting [6]. Inpatient care aims at stabilization and does not aim at regaining healthy weight. In the inpatient unit, the assistance around mealtimes and the ‘therapeutic climate’ are built on CBT-E strategies and comprises individual sessions, psychoeducational group meetings, a physical exercise group and a weekly review meeting between patients and their therapists [27]. During the CBT-E period, patients were admitted if they were unstable and/or if outpatient treatment lacked progress; BMI was not used as a fixed admission criterium. During both periods, all inpatients received outpatients sessions as well; preparatory sessions prior to admission and post-hospitalization sessions aimed at maintaining progress and minimizing the risk of relapse. During CBT-E, outpatients sessions addressed the ED psychopathology as well.

### Implementation of CBT-E

In early 2015, after training all staff of all disciplines, the treatment center transformed the inpatient and the outpatient units into CBT-E based programs. Over a six months period, outpatient therapy groups and day-care programs were phased out. In the outpatient unit, the only psychological treatment offered was CBT-E *Underweight,* focused version. The complete implementation plan, including a description of the CBT-E based inpatient care, has been published previously [28].

### Therapist training

During TAU, 24 psychologists, psychotherapists, psychiatrists and nurse practitioners delivered psychological treatments. Other staff members, 16 in total, consisting of psychiatric nurses, dieticians and a psychomotor therapist, delivered partial treatment interventions within the intensive settings.

Before implementing CBT-E, all staff completed the web based CBT-E training and exam offered by Centre for Research on Eating Disorders at Oxford and worked through the guide [6]. In addition, the staff attended two workshops run by C. Fairburn. Since CBT-E *Underweight* is difficult to master, only after completing around 15 CBT-E *normal weight*, staff members were allowed to offer CBT-E *Underweight* for outpatients after working through the additional digital CBT-E *Underweight* modules. During CBT-E, outpatient treatments were delivered by 13 therapists, in the inpatient unit 11 staff members delivered partial interventions; these 24 staff members also delivered treatment during TAU. Throughout 2015–2017, to maximize adherence, all therapists attended a weekly, two hour peer intervision, supervised by senior therapists. Case load records were used to ensure all patients were discussed regularly. To monitor adherence, audiotaped sessions were randomly reviewed.

#### Cost-effectiveness calculations

The cost-effectiveness analyses were performed from the direct treatment costs, health care provider perspective. For each patient, treatment costs (in euros) were established by multiplying standard Dutch cost prices, index year 2014 [29] by the amount of time spent on outpatient contacts (both contacts directly with patients and contacts concerning patients), by the number of days in day-care and/or number of hospitalization days. Referral costs were not included. The time horizon of this study is from start to end-of-treatment; since this horizon was little over a year for both cohorts, no discounting for future costs/effects was applied. Differences in costs and effects between both cohorts were calculated as difference in cumulative direct costs. Incremental cost-effectiveness ratios (ICERs) were calculated for each bootstrapped sample as ICER = (Costs_CBT-E _− Costs_TAU_)/(Effects_CBT-E _− Effects_TAU_) where effect was the robust effect measure. ICERs were calculated separately for all patients and for the subgroup of outpatients only, to explore the influence of hospitalization costs. The ICERs were plotted on cost-effectiveness planes and used for further calculations. TAU as comparator intervention is positioned in the origin of the cost-effectiveness plane with the horizontal axis indicating differences in effect and the vertical axis differences in costs. The scatter plots of ICERs are divided into four specific quadrants along the horizontal and vertical axis with ICERs in the upper right quadrant indicating CBT-E generating better health gain at additional costs, ICERs in the lower left indicating less health gain from CBT-E at lower costs. While TAU dominates CBT-E in the upper left quadrant, CBT-E dominates TAU with better effects at lower costs in the lower right quadrant. The scatter plot of ICERs in more than one quadrant indicates uncertainty about whether the examined intervention is comparatively cost-effective.

Based on the distribution of the ICERs, cost-effectiveness acceptability curves (CEACs, [30]) were provided. CEACs indicate the probability of CBT-E being more cost-effective than TAU as a function of the willingness-to-pay (WTP) for one additional recovered patient. The WTP for one additional recovered Dutch anorexia nervosa patient is, to our knowledge, yet to be established.

#### Statistical analysis

Statistical analyses were performed with SPSS version 25 and the R statistical computing environment. Group differences for continuous data were expressed as mean difference, categorical data as difference in proportion. Categorical measures between two groups were compared using chi-square or Fisher’s exact tests (as appropriate); to compare continuous data, *t*-tests or Mann–Whitney U tests were used. Differences within groups were compared using paired *t*-tests for continuous data. Statistical significance was defined as α = .05, two-sided, with 95% confidence intervals (CI) to express data uncertainty. To analyze repeated continuous outcomes, linear mixed model analyses were conducted according to a two-level structure (patient and repeated measures). As the proportion of patients receiving inpatient treatment differed between both cohorts, an additional sensitivity analysis was performed including a covariate inpatient yes/no. In addition, differences in weight regain between patients with and without inpatient treatment were analyzed. Cohen’s *d* were used to examine effect sizes.

Clinical outcome analyses were performed on a dataset with observed values and on a pooled imputed dataset.

For missing data, multiple imputation, with 50 imputations for each missing observation, was used, under a missing-at-random assumption; no differences were found between patient groups with and without complete EDE-Q global scores. Analyses were performed first on the imputed datasets separately, and then the outcomes of the 50 imputations were combined using Rubin’s rules [31], using SPSS. A total of 2500 nonparametric bootstrapped samples was extracted from the 50 imputed data sets, with the number of patients per sample equal to the patient numbers in the original dataset. All cost-effectiveness analyses were run on intention-to-treat basis, using a pooled multiple imputed dataset. Unless otherwise indicated, cost-effectiveness findings are based on these imputed data. The Journal Article Reporting Standards [32], the Strengthening the Reporting of Observational Studies in Epidemiology statement [33] and the Consolidated Health Economic Evaluation Reporting Standards statement [34] were used for study design and reporting of the results.

## Results

### Participants flow

**Fig. 1:**
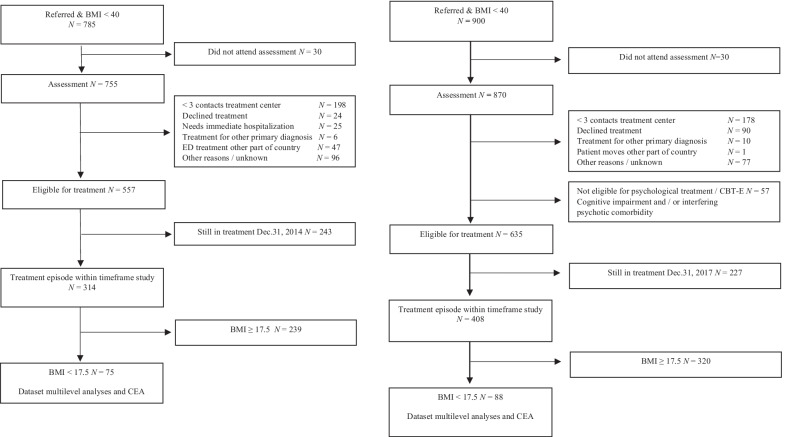
Flowcharts for TAU cohort and CBT-E cohort

The flow of participants for both cohorts are shown in Fig. 1.

### Characteristics of TAU cohort and CBT-E cohort

Baseline characteristics of both cohorts are shown in Table 1, no differences were present on clinical characteristics. In the TAU cohort, 42.4% of patients had an illness duration ≥ 7 years (range 0.5–51 years), in the CBT-E cohort 39.3% (range 0.3–40 years). With regard to BMI under 15, 24.0% in the TAU cohort (lowest BMI 12.5) and 23.9% in the CBT-E cohort (lowest BMI 12.8) were severely underweight.

**Table 1:** Baseline characteristics of TAU cohort and CBT-E cohort

	TAU cohort		CBT-E cohort		*p*
	*N*	*M* (*SD*)	*N*	*M* (*SD*)	
Age, years	75	26.99 (10.85)	88	26.43 (8.73)	.716
Body mass index	75	15.84 (1.15)	88	15.69 (1.16)	.398
Body mass index < 15, *n* (%)	18	24.0%	21	23.9%	.984
Duration eating disorder, years	59	7.90 (9.99)	56	8.55 (9.24)	.718
Gender, *n* (%)					
Male	1	1.3%	4	4.5%	.375
Female	74	98.7%	84	95.5%	
DSM-IV/5 status, *n* (%)					.192
Anorexia nervosa	60	80.0%	77	84.0%	
EDNOS/OSFED	15	20.0%	11	16.0%	
Eating Disorder Examination-Questionnaire					
Global Score	50	3.68 (1.35)	82	3.65 (1.29)	.879
Dietary Restraint subscale	50	3.57 (1.73)	82	3.49 (1.57)	.797
Eating Concern subscale	50	3.17 (1.44)	82	3.22 (1.45)	.855
Weight Concern subscale	50	3.86 (1.54)	82	3.70 (1.56)	.552
Shape Concern subscale	50	4.13 (1.52)	82	4.18 (1.45)	.849
Objective binges, *n* (%)	17	(17/50) 34.0%	32	(32/82) 39.0%	.562
Vomiting, *n* (%)	14	(14/50) 28.0%	26	(26/82) 31.7%	.653
Laxatives, *n* (%)	7	(7/50) 14.0%	11	(11/82) 13.4%	.924
Depression Anxiety Stress Scale					
Global score	54	56.52 (28.79)	81	55.80 (27.95)	.886
Depression subscale	54	20.33 (11.79)	81	20.12 (11.59)	.919
Anxiety subscale	54	14.55 (10.16)	81	14.59 (10.23)	.984
Stress subscale	54	21.63 (10.97)	81	21.09 (9.83)	.765

Data shown as mean (*SD*) unless otherwise indicated

*EDNOS* eating disorder not otherwise specified, *OSFED* other specified feeding and eating disorders

Due to missing data, sample sizes vary across some analyses. When comparing patients with complete measures at baseline and end-of-treatment with patients without complete measures in the TAU cohort, no differences were found with regard to demographics and psychopathology, although patients with complete measures received more treatment sessions (*p* = .022). In the CBT-E cohort, patients with complete measures had higher EDE-Q *global*, *restraint* and *shape concern* scores (respectively *p* = .021, *p* = .025 and *p* = .029) and a longer inpatient stay (*p* = .001). Comparing completers between both cohorts did not show any clinically relevant differences.

Treatment outcomes on observed data on eating disorder and general psychopathology are shown in Table 2.

**Table 2:** Changes in eating disorder (EDE-Q) and general psychopathology (DASS) TAU and CBT-E cohort (observed data)

	TAU cohort							CBT-E cohort							*EMD*, *p*
	Start		End of treatment		*d*	t/χ2	*p*	Start		End of treatment		*d*	t/χ2	*p*	
	*N* start	*M* (*SD*)	*N* EOT	*M* (*SD*)				*N* start	*M* (*SD*)	*N* EOT	*M* (*SD*)				
Body mass index	75	15.84 (1.15)	74	16.9 (1.90)	− .88	− 3.90	.001**	88	15.69 (1.16)	87	18.1 (2.09)	− 1.64	− 10.94	< .001***	1.39, *p* < .001***
EDE-Q															
Global score	50	3.68 (1.35)	26	2.56 (1.64)	.76	3.64	.001**	82	3.65 (1.35)	61	2.41 (1.53)	1.03	9.58	< .001***	− 0.24*, p* = .404
Dietary restraint subscale	50	3.57 (1.73)	26	1.93 (1.85)	1.06	5.67	< .001***	82	3.49 (1.56)	61	1.91 (1.39)	1.26	9.73	< .001***	.004*, p* = .991
Eating concern subscale	50	3.17 (1.44)	26	2.00 (1.71)	.62	2.91	.008**	82	3.22 (1.45)	61	1.99 (1.50)	.95	8.26	< .001***	− 0.25*, p* = .423
Weight concern subscale	50	3.68 (1.54)	26	2.74 (1.81)	.60	2.77	.011*	82	3.70 (1.56)	61	2.57 (1.77)	.80	6.85	< .001***	− 0.15, *p* = . 672
Shape concern subscale	50	4.13 (1.52)	26	3.55 (1.78)	.39	1.79	.086	82	4.18 (1.45)	61	3.16 (1.82)	.76	6.29	< .001***	− 0.52*, p* = .150
Eating disorder behaviour															
Objective binges, n(%), if present	17	(17/50) 34.0%	8	(8/26) 30.8%					(32/82) 39.0%		(27/61) 44.3%				
Episodes/28 days (median, range)		8 (2–140)		13 (8–30)			.763	32	5 (1–90)	27	4 (1–80)			.459	
Vomiting, n(%), if present	14	(14/50) 28.0%	4	(4/26) 15.4%					(26/82) 31.7%		(12/61) 19.7%				
Episodes/28 days (median, range)		16 (1–100)		7 (2–100)			.477	26	13.5 (1–300)	12	4 (1–80)			.066	
Laxatives, n(%), if present	7	(7/50) 14.0%	2	(2/26) 7.7%			1.00	11	(11/82) 13.4%		(5/61) 8.2%			.543	
Episodes/28 days (median, range)		4 (2–28)		17 (6–28)					8 (2–25)	5	3 (1–10)				
Depression Anxiety Stress Scale															
Global score	54	56.52 (28.79)	31	35.74 (25.09)	.67	3.67	.002**	81	55.80 (27.95)	53	43.77 (34.16)	.33	3.48	.001**	7.22, *p* = .120
Depression subscale	54	20.33 (11.79)	31	11.94 (11.71)	.57	2.61	.018*	81	20.12 (11.59)	53	16.42 (14.42)	.26	2.66	.010*	4.22, *p* = .047*
Anxiety subscale	54	14.55 (10.16)	31	8.71 (7.61)	.63	2.99	.008**	81	14.59 (10.23)	53	10.56 (11.10)	.28	2.64	.011*	1.60, *p* = .372
Stress subscale	54	21.63 (10.97)	31	15.10 (8.64)	.57	2.45	.026*	81	21.09 (9.83)	53	16.79 (11.60)	.36	3.27	.002**	1.53, *p* = .425

*EDE-Q* Eating Disorder Examination Questionnaire, *DASS* Depression Anxiety Stress Scale, *EOT* end of treatment, *EMD* estimated mean difference

### Remission

No difference on remission rate between both cohorts was found with 23.1% achieving remission during TAU and 19.7% during CBT-E (*p* = .720)*.* When examining remission rate on the imputed data set, again remission rate did not differ with 9.3% of patients achieving remission during TAU versus 14.6% during CBT-E (*p* = .304).

Eating disorder attitudes as well as general psychopathology improved in both cohorts (all *p* < .001), except for the EDE-Q subscale *shape concern*, which did not improve during TAU. In both cohorts, eating disorder behaviors did not improve significantly, although in the CBT-E cohort, a trend on the decrease of vomiting was found (*p* = .066). Pooled paired sample tests on an imputed dataset showed that in both cohorts, eating disorder attitudes and general psychopathology improved (all *p* < .001).

### Weight regain

With regard to the secondary outcome variable weight regain, findings showed a mean increase during TAU of 1.3 kg/m^2^ (*SD* = 1.6; 95% CI [0.62–2.01]), and of 2.7 kg/m^2^ (*SD* = 1.9; 95% CI [2.2–3.2]). Imputed linear mixed model analyses indicated a comparatively higher increase during CBT-E (*EMD* = 1.33 kg/m^2^, *SE* = 0.29, 95% CI [0.76–1.90], *p* < .001). A difference was found between both cohorts with 17.6% (13/74) of patients reaching BMI ≥ 18.5 during TAU versus 47.1% (41/87) during CBT-E (*p* < .001).

### Admission rate

Admission rate differed between both cohorts; during TAU, fewer patients were admitted (25/75, 33.3%) compared to the number of patients during the CBT-E period (58/88, 65.9%) (*p* < .001). During TAU, mean number of inpatient nights was 64.4 (*SD* = 5.58), higher than the mean number of 44.3 nights (*SD* = 24.0) during CBT-E (*p* = .002).

In both cohorts, inpatients showed more weight regain than outpatients; after accounting for baseline BMI, *EMD* = 0.84 kg/m^2^ (*SE* = 0.30, 95% CI [0.26–1.42], *p* = .005).

As admission rate differed, hospitalization was added as covariate in logistic regression and linear mixed model analyses to account for confounding effects. When including hospitalization as covariate on imputed data, no difference on remission between both cohorts was found (OR = 2.14, 95% CI [0.72–6.38], *p* = .173). When including hospitalization as covariate on imputed data, weight regain was comparatively higher in the CBT-E cohort (*EMD* = 1.33 kg/m^2^, *SE* = 0.29, 95% CI [0.76–1.9], *p* < .001).

### Treatment duration, number of outpatient sessions and day-care duration

The mean treatment duration during TAU of 240 days (*SD* = 147, range 21–763) did not differ from the mean duration during CBT-E of 247 days (*SD* = 115, range 23–686; *p* = .949). The mean number of outpatient sessions during TAU was 14.5 (*SD* = 12.1; range 4–62, median = 11), lower than the mean number of 31.4 sessions during CBT-E (*SD* = 15.6; range 4–57, median = 31; *p* < .001). During TAU, the mean number of days spent in day-care was 28 (*SD* = 21; range 1–77, median = 25), higher than the mean of 7 days during CBT-E (*SD* = 8.6; range 1–44, median = 5.5; *p* = .001).

### Attrition rate for outpatients

For outpatients, dropout rate was defined as having attended fewer than ten treatment sessions, as in the CBT-E *Underweight* this is when the first evaluation takes place. During TAU, attrition rate was higher with 46.1% (18/39) compared to 11.5% during CBT-E (3/26), (*p* = .003). For inpatients, dropout rate was not be established as no minimum duration of overnight stays was defined.

### Treatment costs

Table 3 shows mean direct treatment costs for both cohorts.

**Table 3:** Eating disorder treatment cost categories and differences in costs between CBT-E cohort and TAU cohort

Costs (in euros)	TAU cohort *N* = 75	CBT-E cohort *N* = 88	CBT-E versus TAU
	*M* (*SD*)	*M* (*SD*)	Bootstrapped mean difference [95% CI]
Costs outpatient treatment	2377 (2047)	3634 (1998)	− 1258 [− 1856 to − 615]
Number of outpatient sessions	14.5 (12.1)	31.4 (15.6)	
Costs day-care treatment	1137 (2657)	348 (948)	775 [199 to 1437]
n (%) receiving day-care	18 (24.0%)	26 (29.5%)	
Number of days	28 (21)	7 (8.6)	
Costs inpatient treatment	6483 (10,410)	8827 (8676)	
n (%) receiving inpatient care	25 (33.3%)	58 (65.9%)	
Number of overnight stays	64.4 (5.6)	44.3 (24.0)	− 2366 [− 5224 to 733]
Total costs	9997 (10,467)	12,809 (8649)	− 2826 [− 5672 to 178]

Presented costs are the costs accrued during treatment episode. 95% CI is the 95% confidence interval around the bootstrapped mean difference

With regard to total treatment costs, findings indicated a bootstrapped mean difference of €2826,—(95% CI [− 5672–178]), with higher costs during the CBT-E period.

### Cost effectiveness

**Fig. 2:**
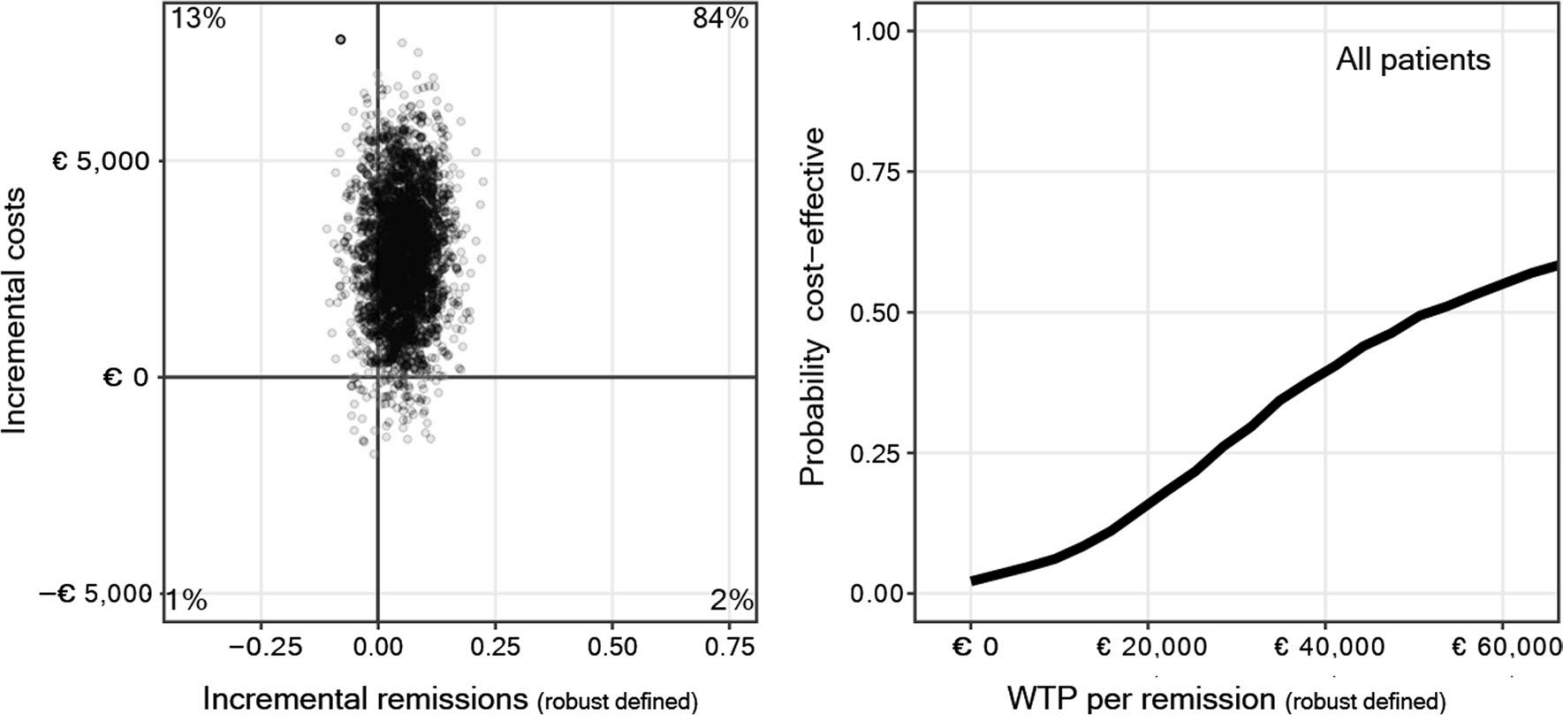
Cost-effectiveness plane and cost-acceptability curve of the base case scenario for all patients with remission as effect parameters

The cost-effectiveness plane for the population of all patients, employing remission (BMI ≥ 18.5, an EDE-Q global score under 2.77 and the absence of binge/purge behaviours) as effect measure, is shown in Fig. 2. The distribution of ICERs in the upper right quadrant indicates a 84% likelihood of CBT-E generating better health gain at additional costs, and a 13% likelihood of less effects at additional costs.

The median ICER is €51,081, indicating a probable preference for CBT-E (> 50% probability of cost-effectiveness) assuming a WTP of €51,081 or more for each additional remission. Examining outpatients only, the likelihood of CBT-E generating better health gain is higher, with an ICER of €11,713, and a CEAC indicating a probability of CBT-E being cost-effective assuming a WTP of €11,713, see Additional file 1: Fig. S3.

## Discussion

This study examined effectiveness and cost-effectiveness for adult anorexia nervosa patients after implementing cognitive behavior therapy-enhanced (CBT-E), a recommended specialized treatment, throughout a routine treatment center offering both outpatient and inpatient treatment. For this purpose, two consecutive cohorts of significantly underweight patients were compared, one receiving regular treatment-as-usual (TAU) between 2012–2014, the next receiving CBT-E between 2015–2017. Both cohorts were seriously affected, with around 24% of patients having a BMI under 15, and around 40% of patients having an illness duration of seven years or longer.

The first main finding is that, when using direct treatment costs in the cost-effectiveness analysis, employing remission as effect measure, the ICER distribution for all patients indicates a 84% likelihood of CBT-E generating better effects compared with TAU, albeit at higher costs. The CEAC indicates a probability of CBT-E being more effective at higher costs compared with TAU, assuming a WTP of €51,081 or more for each additional remission. The WTP for one additional recovered Dutch anorexia nervosa patient is, to our knowledge, yet to be established.

Second, no relevant differences with regard to remission rate and/or clinical outcomes were found between both cohorts, other than on weight regain. After accounting for possible confounding effects of hospitalization, as admission rate differed between both cohorts, findings indicate a comparatively higher weight regain during CBT-E. In both cohorts, anorexia nervosa attitudes improved and in both cohorts, no decrease on bingeing, vomiting and laxatives misuse was found. With regard to those behaviors it is worth noting that the use of the self-report EDE-Q may have overestimated the reporting of binges in both cohorts [35].

During CBT-E, an estimated mean difference of €2826,—higher treatment costs was found. One of the aims within the CBT-E implementation was moving treatment of stable significantly underweight patients away from prolonged intensive outpatient and/or prolonged inpatient treatment, to individual outpatient care. In order to enhance this shift to outpatient treatment, it was decided to stop offering intensive outpatient daycare. At hindsight, this decision may have contributed to both higher outpatient treatment costs due to an increased number of received sessions and higher inpatient costs due to a higher admission rate. As staff felt a considerable number of patients did not response optimally to outpatient treatment, admission rate increased.

In 2020, findings from our treatment center on comparative (cost-)effectiveness after implementing CBT-E for patients with BMI over 17.5 were published [36]. For these patient groups, contrary to the present findings, changing to CBT-E led to a decrease in treatment costs; shortening the inpatient stay from around 13 weeks during TAU to around 8 weeks led to reduced costs as admission rate did not change for the BMI over 17.5 cohorts, combined with a cost reduction due to stopping offering intensive outpatient treatment. When combining both patient groups of BMI over and below 17.5, total direct treatment costs are €19.006 during TAU and €17.739 during CBT-E*.*

The global treatment costs of €12,809,during CBT-E contrast well with internationally reported average treatment costs [37]. The comparatively higher admission rate during CBT-E may be related to staff members mastering CBT-E *Underweight*, in order to treat severely underweight patients on an outpatient basis, combined with available inpatient facilities at hand.

Anorexia nervosa treatments are known for their high dropout rates, as was the outpatient dropout rate during TAU. The found lower outpatient attrition rate during CBT-E may be related to the acceptability of the treatment method as staff is able to commit more outpatients to complete their treatment.

As literature suggests that studies with reported training have a larger effect on weight regain compared to studies without reported training [2], perhaps the structured training and/or structured supervision during CBT-E may be one of the contributing variables to the found difference in weight regain.

Comparing our findings on the CBT-E cohort with other CBT-E anorexia effectiveness studies is somewhat limited because some studies excluded patients with BMI < 15 or offered either solely outpatient or solely inpatient treatment. When comparing the BMI increase of 2.75 (*SD* = 1.9) during CBT-E with 2.77 (*SD* = 1.81) of the Fairburn anorexia nervosa outpatient study, which did not include patients with BMI < 15, findings appear similar [8]. In the considerable more intensive 20-week, solely inpatient studies of Dalle Grave [13, 38] however, a higher mean BMI increase of 4.8 (*SD* = 1.7) was found with 82% respectively 87.3% of adult completers achieved healthy weight after hospitalization, indicating that prolonged inpatient treatment may lead to better weight regain. In the 2013 Dalle Grave study [13] 48.6% had minimal residual eating disorder psychopathology, defined as global EDE score below 1 *SD* above community mean (i.e. < 1.74); in the present study, 59% (36/61) had minimal residual eating disorder psychopathology defined as global EDE-Q score below 1 *SD* above community mean (i.e. < 2.77) (results not shown).. Comparing the 14.6% CBT-E remission rate with outpatient studies including severely and extremely underweight patients, findings appearin line with the 17% and 8.8% respectively of the Turner and Byrne studies [7, 9], but islower than the 55% in the Calugi study [12], although across the studies different definitions of remission were used as the absence of binge/purge behaviors was not always included in the remission definition.

The hypothesis of CBT-E for adult patients with anorexia nervosa being more effective than TAU was not met on the primary outcome measure remission. Weight regain was comparatively higher in the CBT-E cohort. Economic evaluation showed a probability of CBT-E being dominant on remission compared to TAU, albeit at higher costs.

### Limitations

The non-controlled design of this study comes with limitations; although the 24 staff members delivering CBT-E also delivered TAU earlier, therapist fidelity was not systematically assessed. Rather, to monitor and enhance CBT-E adherence, audiotaped sessions were reviewed during intervision. During both periods, no fixed admission criteria were used; the decision to hospitalize patients was based upon clinical judgement and therefore potentially biased. Although in line with similar studies [7, 9] and analyses suggesting that key patient characteristics, program leadership, operating procedures with regard to patients records and/or financing system had not changed, the five year time frame of this study, may be a confounding variable. Due to missing follow up data of particularly the TAU cohort, comparative longer term effects were not examined; a considerable proportion of patients treated during early TAU was already lost to follow up by start of the CBT-E period. Although this study has a non-controlled design, as none of the baseline characteristics differed noteworthy between both cohorts, it was decided to refrain from applying propensity score inverse probability weighting or equivalent approaches*.*

Additional societal and potential concurrent health costs were not measured and valued; as little knowledge is available on direct treatment costs of anorexia nervosa, the purpose in this study was establishing direct costs.

### Strengths

As this effectiveness study did not take place within the context of a, potentially selective, controlled trial, run with a select group of therapists, findings may be generalizable to other routine settings offering mixed inpatient and outpatient treatment for adult patients with anorexia nervosa. By making clinical outcome and actual treatment costs, instead of model costs, public, this study contributes to enhancing transparency, allowing learning from variations found between services, so helping development of more effective health care [39]. This study may contribute to the evidence of the effectiveness of specialized, recommended psychological treatments for anorexia nervosa. In particular, it contributes to the evidence base on (cost-)effectiveness of CBT-E for underweight patients, in combined inpatient and outpatients settings employing a multidisciplinary staff. A growing evidence base may contribute to decision making in clinical practice, may lessen clinical uncertainty and subsequently reduce eclectic treatment approaches [40].

## Conclusions

When comparing one cohort of adult inpatients and outpatients with anorexia nervosa receiving treatment-as-usual, with the next cohort receiving CBT-E, no difference on remission was found. Findings indicate a probability of CBT-E dominating treatment-as-usual, at additional costs, when employing remission as effect measure. After accounting for possible confounding effects of hospitalization as admission rate differed between both cohorts, weight regain was comparatively higher in the CBT-E cohort (*EMD* = 1.33 kg/m^2^, *SE* = 0.29, 95% CI [0.76–1.9], *p* < .001).

The findings of this study may contribute to the knowledge of effectiveness and cost-effectiveness of specialized psychological treatments.

## Supplementary Information

**Additional file 1: Fig. S3.** Cost-effectiveness plane and cost-acceptability curve of the base case scenario with robust remission as effect parameter (outpatients only).

## Data Availability

The datasets used and/or analyzed during the current study are available from the corresponding author on reasonable request.

## Abbreviations

BMI: Body mass index
CEA: Cost-effectiveness analyses
CBT-E: Cognitive behaviour therapy-enhanced
CEAC: Cost-effectiveness acceptability curve
DASS: Depression Anxiety Stress Scale
EDNOS: Eating disorder not otherwise specified
EDE-Q: Eating Disorder Examination-Questionnaire
EOT: End of treatment
ICER: Incremental cost-effectiveness ratio
NICE: National Institute for Health and Care Excellence
OSFED: Other specified feeding and eating disorders
TAU: Treatment as usual
WTP: Willingness-to-pay

## References

[1] Zeeck A, Herpertz-Dahlmann B, Friederich HC, Brockmeyer T, Resmark G, Hagenah U (2018). Psychotherapeutic treatment for anorexia nervosa: a systematic review and network meta-analysis. Front Psychiatry.

[2] Van den Berg E, Houtzager L, De Vos J, Daemen I, Katsaragaki G, Karyotaki E (2019). Meta-analysis on the efficacy of psychological treatments for anorexia nervosa. Eur Eat Disord Rev Rev.

[3] Madden S, Hay P, Touyz S (2015). Systematic review of evidence for different treatment settings in anorexia nervosa. World J Psychiatry.

[4] Hay P, Touyz S, Claudino A, Lujic S, Smith C, Madden S (2019). Inpatient versus outpatient care, partial hospitalisation and waiting list for people with eating disorders. Cochrane Database Syst Rev.

[5] NICE National Institute for Health and Care Excellence. Eating disorders: recognition and treatment. Version 2.0. https://www.nice.org.uk/guidance/ng69.

[6] Fairburn CG (2008). Cognitive behavior therapy and eating disorders.

[7] Byrne S, Fursland A, Allen K, Watson H (2011). The effectiveness of enhanced cognitive behavioural therapy for eating disorders: an open trial. Behav Res Ther.

[8] Fairburn C, Cooper Z, Doll H, O’Connor M, Palmer R, Dalle GR (2013). Enhanced cognitive behaviour therapy for adults with anorexia nervosa: a UK–Italy study. Behav Res Ther.

[9] Turner H, Marshall E, Stopa L, Waller G (2015). Cognitive behavioural therapy for outpatients with eating disorders: effectiveness for a transdiagnostic group in a routine clinical setting. Behav Res Ther.

[10] Signorini R, Sheffield J, Rhodes N, Fleming C, Ward W (2017). The effectiveness of enhanced cognitive behavioural therapy (CBT-E): a naturalistic study within an outpatient eating disorder service. Behav Cogn Psychother.

[11] Frostad S, Danielsen Y, Rekkedal G, Jevne C, Dalle Grave R, Øyvind R (2018). Implementation of enhanced cognitive behaviour therapy (CBT-E) for adults with anorexia nervosa in an outpatient eating-disorder unit at a public hospital. J Eat Disord.

[12] Calugi S, Sartirana M, Frostad S, Dalle GR (2021). Enhanced cognitive behavior therapy for severe and extreme anorexia nervosa: an outpatient case series. Int J Eat Disord.

[13] Dalle Grave R, Calugi S, Conti M, Doll H, Fairburn CG (2013). Inpatient cognitive behaviour therapy for anorexia nervosa: a randomized controlled trial. Psychother Psychosom.

[14] Thompson-Brenner H, Brooks GE, Boswell JF, Espel-Huynh H, Dore R, Franklin D (2018). Evidence-based implementation practices applied to the intensive treatment of eating disorders: summary of research and illustration of principles using a case example. Clin Psychol.

[15] Crow S, Agras W, Halmi K, Fairburn C, Mitchell J, Nyman J (2013). A cost effectiveness analysis of stepped care treatment for bulimia nervosa. Int J Eat Disord.

[16] Le LK-D, Hay P, Mihalopoulos C (2018). A systematic review of cost-effectiveness studies of prevention and treatment for eating disorders. Aust N Z J Psychiatry.

[17] Byford S, Barrett B, Roberts C, Clark A, Edwards V, Smethurst N (2007). Economic evaluation of a randomized controlled trial for anorexia nervosa in adolescents. Br J Psychiat.

[18] Egger N, Wild B, Zipfel S, Junne F, Konnopka A, Schmidt U (2016). Cost-effectiveness of focal psychodynamic therapy and enhanced cognitive-behavioural therapy in out-patients with anorexia. Psychol Med.

[19] American Psychiatric Association (1994). Diagnostic and statistical manual of mental disorders: DSM-IV.

[20] American Psychiatric Association (2013). Diagnostic and statistical manual of mental disorders.

[21] Fairburn CG, Beglin SJ, Fairburn CG (2008). Eating disorder examination questionnaire (6.0). Cognitive behavior therapy and eating disorders.

[22] Aardoom JJ, Dingemans AE, Slof Op ’t Landt MCT, Van Furth EF (2012). Norms and discriminative validity of the Eating Disorders Examination Questionnaire (EDE-Q). Eat Behav.

[23] Lovibond PF, Lovibond SH (1995). The structure of negative emotional states: comparison of the Depression Anxiety Stress Scales (DASS) with the Beck Depression and Anxiety inventories. Behav Res Ther.

[24] De Beurs E, Van Dyck R, Marquenie LA, Lange A, Blonk RWB (2001). De DASS: een vragenlijst voor het meten van depressie, angst en stress. Gedragstherapie.

[25] Mond J, Hay P, Rodgers B, Owen C (2006). Eating Disorder Examination Questionnaire (EDE-Q): norms for young adult women. Behav Res Ther.

[26] Waller G (2016). Recent advances in psychological therapies for eating disorders. F1000Res.

[27] Dalle Grave R, Bohn K, Hawker D, Fairburn CG, Fairburn CG (2008). Inpatient, day patient and two forms of outpatient CBT-E. Cognitive behavior therapy and eating disorders.

[28] Van den Berg E, Schlochtermeier D, Goudriaan A, Dekker J (2017). Implementatie van cognitive behavioral therapy-enhanced in een regulier behandelcentrum voor eetstoornissen. Tijdschrift voor Gedragstherapie.

[29] Zorginstituut Nederland. Richtlijn voor het uitvoeren van economische evaluaties in de gezondheidszorg [Guideline for economic evaluations in health care]. https://www.zorginstituutnederland.nl/publicaties/publicatie/2016/02/29/richtlijn-voorhet-uitvoeren-van-economische-evaluaties-in-de-gezondheidszorg.

[30] Van Hout BA, Al MJ, Gordon GS, Rutten FF (1994). Costs, effects and C/E-ratios alongside a clinical trial. Health Econ.

[31] Rubin BD (1987). Multiple imputation for nonresponse in surveys.

[32] Applebaum M, Cooper H, Kline R, Mayo-Wilson E, Nezu A, Rao S (2018). Journal article reporting standards for quantitative research in psychology: the APA publications and communications board task force report. Am Psychol.

[33] Vandenbroucke JP, Von Elm E, Altman DG, Gøtzsche PC, Mulrow CD, Pocock SJ (2007). Strengthening the reporting of observational studies in epidemiology (STROBE): explanation and elaboration. Epidemiology.

[34] Husereau D, Drummond M, Petrou S, Carswell C, Moher D, Greenberg D (2013). Consolidated health economic evaluation reporting standards (CHEERS)—explanation and elaboration: a report of the ISPOR health economic evaluations publication guidelines task force. Value Health.

[35] Fairburn CG, Beglin SJ (1994). Assessment of eating disorders: interview or self-report questionnaire?. Int J Eat Disord.

[36] van den Berg E, Schlochtermeier D, Koenders J (2020). Implementing cognitive behavioral therapy-enhanced in a routine inpatient and outpatient setting: comparing effectiveness and treatment costs in two consecutive cohorts. Int J Eat Disord.

[37] Stuhldreher N, Wild B, König H-H, Konnopka A, Zipfel S, Herzog W (2012). Determinants of direct and indirect costst in anorexia nervosa. Int J Eat Disord.

[38] Dalle Grave R, Conti M, Calugi S (2020). Effectiveness of intensive cognitive behavioral therapy in adolescents and adults with anorexia nervosa. Int J Eat Disord.

[39] Clark D, Canvin L, Green J, Layard R, Pilling S, Janecka M (2018). Transparency about the outcomes of mental health services (IAPT approach): an analysis of public data. Lancet.

[40] Wonderlich SA, Bulik CM, Schmidt U, Steiger H, Hoek HW (2020). Severe and enduring anorexia nervosa: update and observations about the current clinical reality. Int J Eat Disord.

