# Supplementary material for: Effectiveness and cost-effectiveness of cognitive behavior therapy-enhanced compared with treatment-as-usual for anorexia nervosa in an inpatient and outpatient routine setting: a consecutive cohort study
Source: J Eat Disord. 2022 Jan 6;10:2. doi: 10.1186/s40337-021-00526-1 (PMC8734258; doi:10.1186/s40337-021-00526-1)

**Figure 3***Cost-effectiveness plane and cost-acceptability curve of the base case scenario with robust remission as effect parameter (outpatients only)*


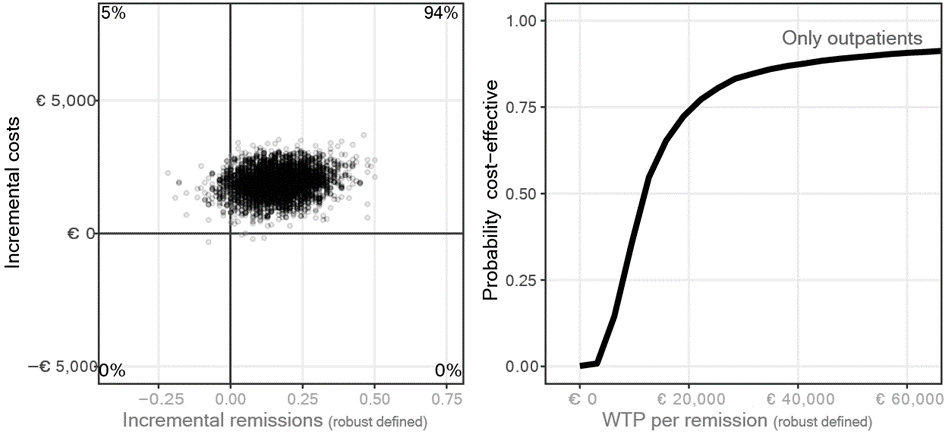

Supplement: Supplementary file 1 — Additional file 1: Fig. S3. Cost-effectiveness plane and cost-acceptability curve of the base case scenario with robust remission as effect parameter (outpatients only). [file 40337_2021_526_MOESM1_ESM.docx]
